# Strategies for Liver Transplantation Tolerance

**DOI:** 10.3390/ijms22052253

**Published:** 2021-02-24

**Authors:** Filip Cvetkovski, J. Mark Hexham, Erik Berglund

**Affiliations:** 1 Research and Development, ITB-Med AB, 113 66 Stockholm, Sweden; filip.cvetkovski@itb-med.com (F.C.); mark.hexham@itb-med.com (J.M.H.); 2 Division of Transplantation Surgery, CLINTEC, Karolinska Institute, 141 86 Stockholm, Sweden

**Keywords:** liver transplantation, tolerance induction, immunosuppression

## Abstract

Liver transplant (LT) recipients require life-long immunosuppression (IS) therapy to preserve allograft function. The risks of chronic IS include an increased frequency of malignancy, infection, renal impairment, and other systemic toxicities. Despite advances in IS, long-term LT outcomes have not been improved over the past three decades. Standard-of-care (SoC) therapy can, in rare cases, lead to development of operational tolerance that permits safe withdrawal of maintenance IS. However, successful IS withdrawal cannot be reliably predicted and, in current prospective studies, is attempted several years after the transplant procedure, after considerable exposure to the cumulative burden of maintenance therapy. A recent pilot clinical trial in liver tolerance induction demonstrated that peri-transplant immunomodulation, using a regulatory T-cell (Treg) approach, can reduce donor-specific alloreactivity and allow early IS withdrawal. Herein we review protocols for active tolerance induction in liver transplantation, with a focus on identifying tolerogenic cell populations, as well as barriers to tolerance. In addition, we propose the use of novel IS agents to promote immunomodulatory mechanisms favoring tolerance. With numerous IS withdrawal trials underway, improved monitoring and use of novel immunomodulatory strategies will help provide the necessary knowledge to establish an active liver tolerance induction protocol for widespread use.

## 1. Introduction

**Figure 1:**
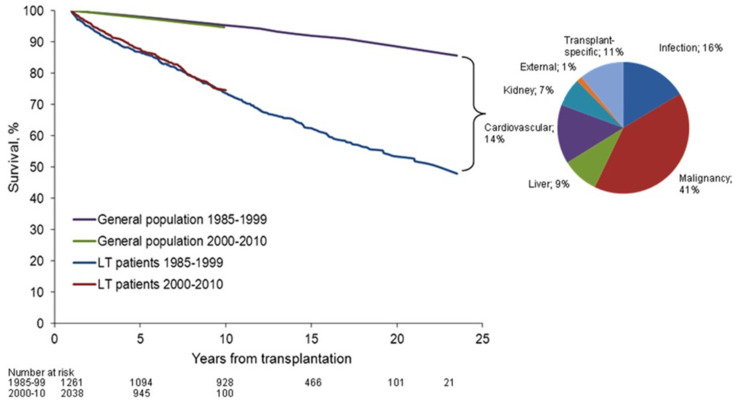
Patient survival rate after liver transplantation compared to general population (from Åberg et al. [3]). Overall patient survival rate beyond the first year post-liver transplantation has not been improved from the 1980s to the 2000s. Pie chart describes the distribution of cause-specific excess mortality among liver transplantation recipients. Elevated standardized mortality rates in liver transplantation patients were evident for infection, malignancy, liver disease, and kidney disease, reflecting the comorbidities of long-term immunosuppression therapy. Reprinted with permission from ref. [3]. Copyright 2015 Wiley. Copyright 2014 by the American Association for the Study of Liver Diseases.

**Figure 2:**
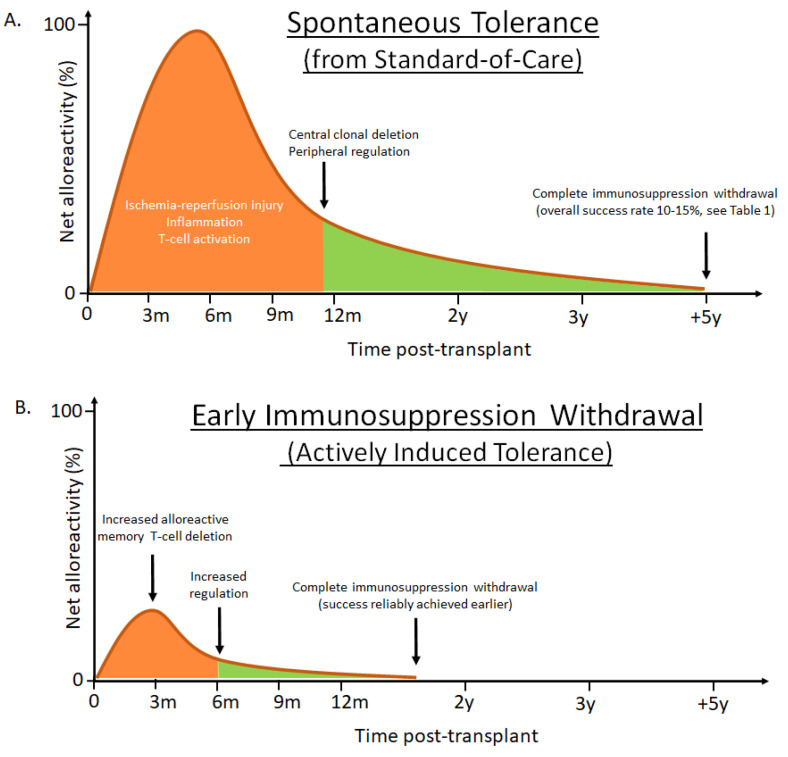
Conceptualized liver transplantation outcomes using either standard-of-care immunosuppression or enhanced tolerance induction strategies. (**A**) Successful immunosuppression withdrawal can, at least in part, be explained by the balance between early triggered alloreactivity (orange) and a gradually re-establishing tolerogenic liver environment (green). When immunosuppression withdrawal is attempted 1–2 years post liver transplantation success is rare, likely due to activation of large numbers of alloreactive memory T-cells [7], enhanced by post-surgery inflammation. After the initial insult has subsided, the natural tolerogenic influence of the liver can contribute to clonal deletion and peripheral regulatory control of alloreactive T-cells. The chances of achieving spontaneous tolerance are highest several years (5–7 years) post-transplant, at a time when significant drug toxicities have accumulated. In addition, among patients considered for withdrawal, several develop eventual rejections and there are currently no validated biomarkers to predict which patients will remain rejection-free [8,9]. (**B**) The hypothesis that enhanced peri-transplant immunomodulation can diminish the level and duration of alloreactivity, through increased deletion of alloreactive T-cells and increasing regulation, to offer reliable and active tolerance induction, has been shown in a proof-of-concept clinical trial by adding cyclophosphamide and regulatory T-cells in living donor liver recipients [5,6]. The strategy allowed complete immunosuppression withdrawal in all non-autoimmune recipients by 18 months post-transplant. Although the patient cohort was limited in size, none of the successfully weaned patients developed subsequent acute or chronic rejections during the study period. It is conceivable that targeted biologics fulfilling similar immune modulatory functions could replace the infused cellular product used by Todo and colleagues, thereby significantly simplifying the strategy and allow widespread use. Net alloreactivity zero represents threshold of successful immunosuppression withdrawal.

Transplantation is the life-saving procedure for end-stage liver disease of various etiologies. The number of annual liver transplants is about 7000 in Europe [1] and 8000 in the US [2]. Early challenges with high rates of acute graft rejection were overcome with the introduction of potent IS regimens, which are largely still in use today. However, long-term improvements in LT graft and patient outcomes have been hampered by the cumulative burden of maintenance IS. The consequences of life-long IS therapy are evident in elevated mortality rates in LT patients for infection, malignancy, cardiovascular events, renal disease, and the incomplete preservation of liver function, when compared with the general population [3] (Figure 1). Alternatives to chronic IS therapy have been developed for other solid organ transplants using tolerance induction, a peri-transplant regimen that actively promotes tolerance of the donor allograft in the recipient, with the goal of safe and complete IS withdrawal [4]. Recent proof-of-concept clinical data suggests that early IS withdrawal is also possible following LT through active tolerance induction [5,6]. Herein we explore the current status of liver transplant tolerance strategies. We hypothesize that targeted peri-transplant immunomodulation may diminish the level and duration of alloreactivity to induce tolerance and allow complete IS withdrawal early post-LT, to reduce the long-term toxicities of chronic IS and improve patient outcomes (Figure 2).

## 2. Spontaneous Operational Liver Tolerance Is a Rare Outcome Post-Transplant on Standard-of-Care Immunosuppression

Analysis of patient survival and quality of life in LT recipients indicates an absence of significant clinical progress in long-term outcomes over the past three decades. In the US, the one-year patient survival in 2015 compared to 1986 has improved markedly, from 66% to 92% [10]. This improvement in short-term outcomes contrasts with stagnant patient survival rates beyond the first year post-LT, with high mortality driven by malignancies, non-rejection graft failure and infection. Notably, the prevalence of malignancies and infections has not been affected by the incremental advances in IS (including the shifts from cyclosporine to tacrolimus (TAC) and from azathioprine to mycophenolic acid), suggesting that toxicity is less associated with the specific drugs, but rather to long-term use of chronic IS [10]. In addition, renal failure requiring maintenance dialysis or renal transplantation has been documented at 36 months post-transplant in 17% recipients of non-renal organs, including liver [11]. This renal impairment, likely due to calcineurin inhibitor use, was significantly associated with a 4.55-fold increase in the relative risk of death. To further elucidate the toll of life-long SoC IS therapy, a study of Nordic LT recipients sought to compare the overall and cause specific mortality to the general population [3]. Overall patient survival beyond the first year post-LT remained unchanged from the 1980s to the 2000s, as patients faced a 2.4-fold increased risk of death, and a 5.8-fold increased risk of premature death (before age 75), when compared with the general population. Specifically, elevated standardized mortality rates in LT patients were evident for infection, malignancy, liver disease, and renal disease (Figure 1). Studies on patient cohorts across Europe [1] and the US [12] have reached similar conclusions. In contrast, IS-free LT patients have improved cardiovascular risk factors, renal function, and metabolic parameters, demonstrating the negative impact of SoC on morbidity and mortality [13]. There is thus an unmet need to improve long-term IS maintenance therapy, develop more selective anti-rejection agents that reduce off-target toxicity. The liver has been described as a more tolerogenic graft compared to other organs. In rodents and pig animal models, a liver graft can be accepted spontaneously by the host without administration of IS [14,15,16,17]. In humans, liver allografts can promote immunoprotection of other co-transplanted organs such as a heart or kidney [18,19,20,21]. However, the mechanisms behind the tolerogenic liver effects are insufficiently understood to be reliably translated into the clinic. In addition, although human LT recipients receiving SoC can develop a spontaneous state of operational tolerance that allows safe complete IS withdrawal [22], characterized by a lack of harmful immune responses towards the graft, such outcomes are relatively rare in human trials (Table 1). It is important to note that the encouraging success rates presented are from groups of highly selected recipients who have had stable blood chemistries and clean liver biopsies for several years post-transplant. The probability of successful IS withdrawal increases later post-transplant, although at that time the net impact of chronic IS has increased significantly [23]. While spontaneous tolerance can lead to IS withdrawal among a subset of liver recipients, the long-term outcomes of the IS-free liver grafts vary. Insufficient IS has been associated with an increased incidence of liver fibrosis [24,25,26,27]. Other studies have reported no major benefits to the liver in IS-free patients [28], or have found evidence of chronic injuries in protocol biopsies from otherwise stable recipients [29]. Chronic allograft injury may be the result of ongoing low-grade inflammation with contributions from donor-specific antibodies (DSA), as well as several non-HLA antigens associated with fibrosis [8,30,31,32,33,34,35]. While improved diagnostics in the future will shed more light on allograft health and injury, it is unlikely that the current SoC will offer a broadly applicable and reliable path towards true operational liver tolerance (defined as discontinuation of all IS for at least one year while maintaining stable allograft status).

## 3. Memory T-Cells Are the Main Mediators of Allograft Rejection

Immunological tolerance is maintained through two major mechanisms, identified as central and peripheral tolerance. Central tolerance involves the deletion of self-reacting T lymphocytes during maturation in the thymus and shapes the immune repertoire to avoid the development of autoimmune disease. Self-reactivity is, however, not completely absent in the periphery of healthy organisms, and it is mainly through suppression by Tregs, a key component of peripheral tolerance, that tolerance is preserved. Both arms of immunological tolerance have been studied in an effort to allow acceptance of allograft tissues in organ transplant recipients [49]. Preparative regimens for active tolerance induction are essential to overcome the barrier against non-self, as the human immune repertoire contains a high frequency of alloreactive T-cells [50]. Both naïve and memory T-cell subsets found in the peripheral blood of healthy human subjects can proliferate in response to alloantigens in the mixed lymphocyte reaction (MLR). Effector memory CD8^+^ T-cells pose a particular threat towards transplanted organs with rapid expression of IFN-γ and cytotoxic molecules upon allostimulation, mediated by lower co-stimulation requirements for re-activation such as, for example, reduced CD28 expression [51]. The enhanced memory CD8^+^ T-cell barrier both in the periphery and in the liver allograft has been described in a non-human primate model attempting to induce early liver tolerance [7]. Functionally active memory CD8^+^ T-cells have a low activation threshold, high proliferative capacity, can track to tissues, constitute about 40–50% of T-cells, and express high levels of CD2 [52,53,54,55,56,57,58,59].

Clinical observations within cohorts of kidney and lung transplant recipients have revealed that the frequency of donor-reactive T-cells correlates with the risk of post-transplant acute rejection [60,61]. While the exact specificity and clonal composition of alloreactive memory T-cells is yet to be elucidated, recent studies utilizing high-throughput T-cell receptor (TCR) sequencing illustrate the potential value of studying the dynamics of T-cell specificity in transplantation [62,63]. A study tracking the fate of donor-reactive TCR β sequences in a cohort of human liver allograft recipients receiving SoC induction and maintenance therapy measured TCR β clonality pre- and post-transplant [64]. A reduction from pre-transplant levels of donor-reactive TCR β sequences was found following transplantation and was stable up to 3 years post-transplant. While approximating the number of donor-reactive clones could not be used as a marker to prospectively identify patients that later developed operational tolerance, the low repertoire turnover suggests a peri-transplant window of opportunity for a sustained reshaping of the immune repertoire. Future studies that can distinguish between different T-cell subsets, and gain access to graft biopsy samples will help clarify the potentially unique repertoire dynamics found in liver transplantation. Targeted control of memory T-cell responses therefore remains an attractive approach of novel liver induction strategies.

## 4. T-Cell Depletion as a Strategy for Tolerance Induction

Tolerance induction therapy is administered peri-transplant to prevent acute rejection and to facilitate weaning of long-term maintenance IS. Past approaches employing T-cell depletion and/or immunomodulation have not shown consistent efficacy in liver tolerance induction. However, the development of promising new biologics with novel mechanisms of immunomodulation justifies a re-evaluation of previous attempts at tolerance induction [65,66].

Muromonab-OKT3, a mouse monoclonal antibody specific against CD3 on the surface of human T cells, was the first therapeutic antibody used in solid organ transplantation to reverse acute graft rejection and the first monoclonal antibody approved for human therapy [67,68,69]. During OKT3 administration, T cells are eliminated through antibody-mediated phagocytosis or made unresponsive by TCR internalization [70]. However, the interaction of OKT3 with CD3 leads to transient polyclonal T cell activation and consequent life-threating systemic release of cytokines (so called “cytokine storm”) [71,72,73]. Ultimately, the increased risk of infection and lymphoproliferative disease [74,75], the development of drug sensitization [69], and the lack of long-term benefits to patient survival or allograft health associated with OKT3 therapy [76], led to a decline in use, OKT3 manufacturing was discontinued and the drug was withdrawn from the market.

Rabbit antithymocyte globulin (ATG-G, manufactured by Genzyme) is a purified mixture of polyclonal IgG antibodies raised in rabbits against human thymocytes and was originally introduced as an immunosuppressant for the prevention or treatment of kidney allograft rejection [77]. ATG contains a large diversity of antibodies directed against a range of molecules involved in lymphocyte activation, co-stimulation, and adhesion. Therefore ATG-G may simultaneously exert inhibitory and mitogenic effects upon T-cells [78,79,80]. Many of the first case studies of successful tolerance induction combined ATG-G for depletion or inactivation of T-cells with a range of xenobiotics, steroids or cell-based therapies. The first successful minimization of maintenance IS was reported in a small group of patients that received a single pre-transplant dose of ATG-G prior to liver transplantation [81]. This use of ATG-G allowed the lowering of TAC monotherapy maintenance doses around 6 months post-transplant. While important as a proof-of-principle study, the lack of detailed description of the state of the graft or the lymphocyte profile in circulation precludes analysis of the ATG-G mechanism of action. Complete withdrawal of maintenance IS in two liver transplant recipients was reported using a conditioning regimen with cyclophosphamide (CP) and ATG-G, followed by infusion of purified donor CD34^+^ stem cells [82]. IS was discontinued at 90 and 28 days posttransplant, in these two patients respectively. Subsequent studies successfully exchanged CP for the combination of steroids and rapamycin [83], however, the stem cell infusion appeared to be essential for achieving tolerance [84]. Patients pretreated with ATG-G without the cellular treatment developed acute rejection characterized by an increase of blood CD8^+^ T-cell counts upon IS withdrawal. It was proposed that IL-7-driven homeostatic expansion of memory CD8^+^ T-cells accounted for the resulting acute rejection. The inability of ATG-G to control donor-specific memory CD8^+^ T-cells was confirmed by others [85,86], suggesting incomplete induction of immunological tolerance. Ultimately, the small number of treated patients in the above studies, the short post-transplant follow-up, and the use of suboptimal immune monitoring did not provide sufficient data to describe the precise role of ATG-G in tolerance induction. A second similar ATG product, termed ATG-F (manufactured by Fresenius), is also used in transplantation and this product was also generated by immunization of rabbits but using a different immunogen, namely the Jurkat T-cell line in contrast to the human thymocytes used for the Genzyme ATG. A randomized controlled trial with the primary objective to reduce conventional IS compared peri-transplant Fresenius ATG (ATG-F) combined with low dose TAC with standard TAC in liver transplant recipients [87]. Investigational therapy comprised ATG-F followed by low dose TAC monotherapy that decreased starting 3 months after transplant. The trial was interrupted prior to reaching the 12 month primary end point, as the ATG-F regimen was associated with an increased rate of acute rejection. Although ATG-F treatment promoted the generation of FOXP3^+^ Tregs and depleted naïve T-cell subsets, effector memory CD8^+^ T-cells were increased compared to SoC. In conclusion, these early clinical observations demonstrated that early IS withdrawal or minimization is possible after liver transplantation with a regimen including non-myeloablative conditioning and a donor stem cell infusion.

Alemtuzumab (Campath-1H), a monoclonal antibody against the cell surface glycoprotein CD52, has been used as a lymphocyte-depleting induction therapy reagent in several LT trials [88,89]. Studies administering alemtuzumab for IS withdrawal or minimization found that maintenance IS could be reduced with decreased rates of acute rejection compared to conventional therapy, however, safety complications following virus reactivation and the failure to reach complete IS weaning argue against additional use of this drug in LT patients [90,91,92,93]. Notably, when used as a tolerance induction reagent in kidney transplant studies, alemtuzumab failed to delete a subset of activated and/or memory T-cells, suggesting that it is not able to overcome the memory T-cell barrier to achieve tolerance [94]. Mechanistically, both alemtuzumab and ATG cause immune activation before depletion, which likely contributes to their inability to control the more resistant effector memory responses long-term [66]. Therefore, using an immunomodulatory biologic that instantly blocks immune activation while selectively depleting effector memory cells may have a higher chance of successfully inducing liver tolerance [66,95].

## 5. Biomarkers of Immune Tolerance in Liver Transplantation

Monitoring immunological parameters in the graft or peripheral blood of LT patients may lead to a greater understanding of the mechanism of tolerance induction. Current attempts focus on (1) identifying tolerogenic cell populations that are more prevalent in operationally tolerant recipients than in patients remaining on IS, or (2) defining host immune signatures that predict the success of IS weaning protocols. Naturally occurring Tregs are essential for maintenance of self-tolerance and represent the most closely investigated tolerogenic cell population in transplantation. Tregs mediate their immunomodulatory function via several cell surface protein interactions and downstream signaling systems. CTLA-4 acts as a high affinity ligand of CD80/CD86 to limit the activation of T cells by depriving them of CD28 co-stimulation [96]. Similarly, constitutive high expression of CD25 (IL-2 receptor subunit) on Tregs suppresses IL-2-dependent proliferation of nearby cells [97]. Additionally, Tregs carry surface enzymes CD39 and CD73 that convert proinflammatory adenosine triphosphate into immunosuppressive adenosine [98]. Soluble factors found to be important in the control of host immune responses include Treg-derived inhibitory cytokines TGF-β, IL-10, and IL-35 [99], as well as cytolytic granzymes and perforin capable of inducing apoptosis of targeted effector cells [100]. However, understanding of the most relevant in vivo mechanisms of Treg function in transplantation is limited. Early descriptions of the mechanism of tolerance in IS-free LT recipients investigated donor-specific reactivity of peripheral blood mononuclear cells (PBMCs) after weaning and compared that to the pre-transplant state using the MLR [101]. The development of donor-specific hyporeactivity and downregulation of IFN-γ suggested active suppression of the T-cell response. Notably, depleting Tregs from the MLR diminished but did not break tolerance, suggesting multiple mechanisms of suppression in operationally tolerant LT recipients [102]. Interest in Tregs has remained, as phenotyping of PBMCs in such operationally tolerant LT recipients found an increase in frequency of CD4^+^CD25^high^ T-cells compared to transplant recipients on IS [103]. However, given the length of time post-transplant (mean 9 years) and long duration of weaning (mean 4 years) when the phenotyping was performed, the kinetics of the development are unclear. A longitudinal study of peripheral blood after LT found a reduction of Tregs at three months post-transplant, regardless of tolerance status, and that rejection episodes were associated with low Treg frequency pre- and one year post-transplant [104]. Still, since no graft biopsy samples were analyzed, changes observed in the periphery might not correlate with immunoreactivity in the graft, reflecting the importance of protocol biopsies in novel liver tolerance trials. A follow-up study by the same group demonstrated that the accumulation of Tregs was also taking place in the graft, however since a subset of tolerant patients lacked intra-graft Tregs it is uncertain how much of the tolerant state can be attributed to this one cell population [105]. This point is further highlighted in a prospective study wherein the number of patients with potentially favorable signs for operational tolerance was overestimated when only Treg frequency/cell count were used as tolerance predictors [106]. Nevertheless, an increased frequency of Tregs continues to be associated with a positive prognosis post LT. In particular, CD62L^high^ Tregs, as well as bulk CD4^+^CD25^+^ Tregs have been associated with operational tolerance post-LT [40,107]. Despite these reports, the dynamics of Treg frequency and cell count are incompletely characterized, precluding the use of this as a marker for predicting or monitoring IS withdrawal. For example, a longitudinal assessment of operationally tolerant LT recipients at timepoints before weaning and at 1 and 3 years after complete IS withdrawal highlighted a number of unexplained findings and a highly dynamic state of multiple T-cell subsets [108]. Enrichment of Tregs was found to correlate with an upregulation of immune activation markers in graft biopsies at one year, followed by a drop to pre-weaning Treg levels at the 3 year timepoint post-LT. In contrast, the frequency of Tregs in peripheral blood was on a consistent downward trajectory throughout the duration of the study. Thus, it cannot be excluded that immunological activity surrounding the graft evolves over a number of years post-IS withdrawal. More recently, the short-term kinetics (days 7 and 30 post-transplant) of peripheral Treg frequency were assessed as a predictive value for acute rejection within the first 6 months post-LT [109]. Frequencies of total and activated Tregs at D7 were found to be lower in recipients with either suspected or biopsy-proven acute rejection, suggesting that Treg frequency alone could be considered as a biomarker for rejection. Other reports have demonstrated the incidence of tolerogenic dendritic cells (DC) and gamma delta (γδ) T-cells in operationally tolerant patients. Both IS-free and LT recipients undergoing weaning harbored an increased ratio of CD11c^−^CD123^hi^ to CD11c^+^CD123^−/lo^ DCs in the periphery, when compared to patients receiving maintenance therapy [110]. More recently, a prospective trial of IS withdrawal found increased circulating tolerogenic DCs (CD11c^+^ILT3^+^ILT4^+^) in the tolerant compared to non-tolerant recipient groups [9]. Interestingly, graft biopsies did not confirm observations in the circulation as enrichment of MHCII^+^ILT4^+^CD11c^+^ DCs was described in the non-tolerant group. Alterations to the γδ T-cell compartment have been associated with operational tolerance in multiple studies [46,103,107,111]. However, it has also been suggested that incidence of certain γδ T-cells is driven by systemic viral infections [112,113]. Recently, in vitro experiments have described a role for virus-specific cells in suppression of alloresponses, which provides a possible explanation for the prevalence of γδ T-cells in operationally tolerant as well as hepatitis C virus-positive LT recipients [114]. The functional significance of DCs and γδ T-cells in the development of tolerance requires further investigation. Exploratory high-throughput gene expression studies have been conducted to identify molecular signatures predictive of successfully achieving operational tolerance in patients undergoing IS withdrawal. Liver tissue samples prospectively taken from operationally tolerant and non-tolerant recipients before initiation of drug minimization differed in the expression of iron metabolism genes [115]. The intra-graft measurements were supported by corresponding differences in serum concentrations of hepcidin and ferritin between tolerant and non-tolerant patients. Importantly, the liver biopsy-based gene signature was more predictive of successful IS discontinuation than the blood-based predictors. The predictive power of the liver-biopsy based gene signature was since confirmed in another separate adult LT cohort, however, a more recent pediatric LT trial could not predict the success of IS withdrawal using these iron metabolism genes [8,9]. Non-invasive biomarkers, such as the presence of DSA, can improve allograft health monitoring for subclinical injury, although their predictive capacity is currently limited and needs to be validated in independent studies [116,117]. Thus, there are currently no clinical or serological biomarkers that are considered predictive of operational tolerance [8,118,119]. In summary, more longitudinal and frequent analyses utilizing broader screening approaches (as used in [64,120,121]) may be required to understand the role of Tregs and other immune mechanisms in achieving immunological tolerance.

## 6. Novel Immunoregulatory Strategies for Active Liver Tolerance Induction

A recent encouraging proof-of-concept Phase I/II trial performed by Todo and colleagues in Japan demonstrates, for the first time, the possibility of successful active clinical liver tolerance induction [5,6]. The regimen was based on a non-human primate (NHP) kidney transplant study where tolerance was achieved by infusion of a suppressive cellular product (prepared by ex vivo co-culturing of recipient and donor PBMCs in the presence of monoclonal antibodies against the costimulatory molecules CD80/86) [122]. After 13 days in culture, the recipient CD4^+^ T-cells upregulated CD25 and CTLA-4 expression, suggesting an enrichment of immunoregulatory lymphocytes, however the cultured mix comprised predominantly CD25^−^CD4^+^ T-cells and traces of non-lymphocytes. In addition to receiving the cellular infusion on post-operative day (POD) 13, recipient animals were splenectomized in order to collect autologous cells to generate the tolerizing cellular product, and given CP on POD 5 to deplete alloactivated lymphocytes. Control groups were included to evaluate the relative contributions of the CP and cellular therapies although no non-splenectomized animals were included, raising questions about the need for splenectomy. However, in a separate preclinical tolerance induction regimen splenectomy was found to be a necessary element, which may provide some mechanistic support to the use of splenectomy by Todo et al. [123,124]. Nonetheless, despite lacking some data on the relative contributions of splenectomy, cellular product infusion and lymphocyte depletion with CP in the development of tolerance, the study was translated to the clinic with impressive results [5,6]. The trial tolerance induction regimen mirrored the NHP design. All trial participants were splenectomized, received adult living donor liver transplants, cellular product, CP and were then treated with conventional IS maintenance therapy. Weaning of IS was initiated at 6 months and successfully completed by month 18 post-transplant in seven out of ten patients. These tolerant patients have now been IS-free beyond 6 years [125]. Throughout the patient follow-up, normal liver function was maintained, and no development of immune activity or fibrosis was observed in histological assessment of biopsy samples [6]. Interestingly, circulating Treg frequency in the tolerant patients was dynamic and failed to follow a clear trend. In contrast, the remaining three non-tolerant recipients who were transplanted because of autoimmune liver diseases, developed mild rejection episodes during weaning and resumed conventional low-dose IS. Observations regarding the suppressive potency of the cell product pre-infusion during MLR, as well as the frequency of circulating Tregs during the attempted weaning, were comparable between the non-tolerant and IS-free patients. Taken together, this pilot study demonstrates the potential of alloreactive T-cell depletion and immunoregulation to induce long-lasting tolerance early post-LT and is a major clinical achievement. Even though the protocol was developed in an MHC-mismatched NHP kidney transplant model, when the same regimen was applied clinically to living-donor kidney recipients, half of the patients showed signs of rejection within about the first year post-transplant. The high rejection rate raises concerns about the effect or duration of the Treg product in the renal transplant population and also underscore the relative tolerogenicity of liver as compared to kidney [126].

One hurdle to implement the above Treg enrichment protocol for the successful liver trial outside of Japan has been the unapproved anti-CD80/CD86 mAbs used in the ex vivo co-culturing step to generate the cellular product. However, this may have been addressed by showing that the two mAbs can be replaced with belatacept and still generate a cellular product that may be sufficiently similar to induce similar clinical results [127]. Clinical studies to generate data using this updated manufacturing process are in progress. Another hurdle is that the unpurified infused cell product contains non-Treg populations (other T-cells subsets, as well as NK and myeloid cell populations), preventing a precise description of the composition of the cellular product. From a good manufacturing practice perspective this poses a challenge of how to define process and release criteria for the cellular product, including phenotypic composition and potency assay assessment, which will need to be addressed for a potentially marketed product [128]. Finally, performance of the splenectomy procedure, combined with CP and cellular infusion may be required to achieve tolerance, and their relative contributions to achieving tolerance are not yet known. NHP studies may in this regard offer additional insight into the mechanisms of tolerance induction in LT. A clinically established strategy for tolerance induction has been demonstrated in recipients of combined kidney and bone marrow cell transplantation (CKBMT) [4], relying on early Treg-mediated peripheral tolerance and long-term deletion of anti-donor T-cells (central tolerance) [129,130,131]. In a study of NHP to translate the CKBMT protocol to LT, the recipients rejected the graft rapidly after IS withdrawal despite developing multilineage mixed chimerism (MC) [7]. The rejections were associated with expansion of intra-graft and circulating effector memory CD8^+^ T-cells. Simultaneously, peripheral Treg cell numbers remained unchanged post-transplantation. Similar observations of CD8^+^ T-cell expansion in LT clinical trials and comparable NHP models demonstrate the necessary enhanced suppression of memory lymphocytes in LT tolerance induction [132,133]. Whereas at least transient mixed hematopoietic chimerism is the only method that has induced transplantation tolerance in both animals (mice, pigs, primates) and humans in kidney transplantation [134], mixed chimerism has so far been insufficient to allow early IS withdrawal in LT [7]. It can however not be ruled out that MC level, duration and composition may still be important for a successful liver tolerance outcome, and further studies are required. In SoC LT, mixed chimerism is transiently seen in a large portion of recipients through the outflow of passenger lymphocytes [135,136]. With the addition of anti-CD2 mAb treatment to control memory T-cells it has been shown that all liver transplanted NHPs develop MC, even without infusion of donor bone marrow cells, which was associated with improved long-term graft survival after complete IS withdrawal [137,138]. Tolerance induction using anti-CD2 as a key element has also been successfully applied clinically with transient mixed chimerism in HLA-mismatched kidney transplant recipients without long-term maintenance IS [4,139]. Mechanistically, an anti-CD2 mAb offers targeted effects ideal for supporting tolerance induction, including: effective and selective depletion of effector memory T-cells in the circulation and target tissues, enrichment of functional donor-specific Tregs in vivo, and co-stimulation blockade [65,95,140,141,142]. This mechanistic profile may prove superior to ATG and alemtuzumab [66], which have been the T-cell depleting agents used in previous LT tolerance studies [87,93]. It is therefore conceivable that the Treg infusion performed as part of the successful clinical liver tolerance induction protocol by Todo and colleagues [5,6] could be replaced by the clinically safe anti-CD2 biologic siplizumab, with the potential to greatly simplify the treatment regimen. A clinical trial application evaluating such replacement is underway.

Encouragingly, at least six other human liver tolerance trials are ongoing that aim to increase immune regulation through Treg administration, with or without additional alloreactive T-cell depletion with CP (Figure 2B, Table 2). In the field of advanced Treg therapies overall there are currently more than 50 clinical trials in progress not only in transplantation but in GvHD and autoimmune indications as well [143]. In addition, the ONE trial is a platform trial approach to evaluate seven regulatory T cell strategies within the same trial [144]. Read-outs from these trials will be expected in the coming years, holding the promise that additional approaches will prove to be safe and efficacious.

## 7. Conclusions

The recent clinical advancements have shown that the enhanced memory T-cell barrier early after liver transplantation can be overcome, and that long-term operational tolerance can be established after LT with an acceptable safety profile, at least among patients with underlying non-autoimmune liver diseases. This is sufficient reason to be optimistic that there is a path towards active liver tolerance induction. With numerous IS withdrawal liver trials underway using similar induction principles, there is an increased chance that an optimized regimen will evolve that can withstand the safety and efficacy scrutiny in larger patient cohorts, ultimately allowing widespread use. Future trials will need to show that the long-term clinical benefits of IS-freedom (including patient and graft survival, IS-related complications, quality-of-life as well as healthcare costs) definitively and significantly outweigh the risks of initial tolerogenic regimen and IS.

## Figures and Tables

**Table 1:** Spontaneous operational liver tolerance. Multi-center clinical trials of standard-of-care immunosuppression withdrawal.

Investigator/Trial	Screened Patients	Attempted ISW	Successful ISW	Presented Success Rate	Overall Success Rate *	Ref.
Mazariegos	NA	95	18	19%	NA	[36]
Devlin/Girlanda	NA	18	2	11%	NA	[37,38]
Pons, 2003	NA	9	3	33%	NA	[39]
Pons, 2008	490	12	5	42%	1%	[40]
Eason	340	18	1	6%	<1%	[41]
Tryphonopoulos	NA	104	20	19%	NA	[42]
Tisone	NA	34	8	24%	NA	[43]
Assy	NA	26	2	8%	NA	[44]
de la Garza	138	24	15	63%	11%	[45]
Feng/iWITH (NCT01638559)	2909	88	33	38%	1%	[8]
Levitsky/NCT02062944	1255	15	8	53%	1%	[9]
Shaked/A-WISH (NCT00135694)	286	77	10	13%	3%	[28]
Bohne/NCT00668369	130	34	17	50%	13%	[46]
Feng/WISP-R (NCT00320606)	129	20	12	60%	9%	[47,48]
Benitez/ NCT00647283	500	102	41	40%	8%	[23]

* Overall success rate is derived using the total number of patients screened for each study, where given, as the denominator.

**Table 2:** Trials involving adoptive Treg therapies to actively induce liver transplantation tolerance.

Centre/Trial	Study Phase	Enrolled	Cell Source	Immunosuppression/ Weaning	Outcomes	Ref.
Hokkaido University Hospital, Japan Tolerance induction by a Treg cell therapy in LDLT. (UMIN-000015789)	Phase I/II	10	Autologous donor-specific Tregs. Generated by co-culturing donor (irradiated, thawed) and recipient (fresh) PBMCs, collected via leukapheresis, for 2 weeks under an umbrella of anti-CD80/CD86 mAbs	CP (40 mg/kg) dosed POD 5, cells infused POD 13. SoC MMF and steroids d.c. at 1 month post-tx. CNI tapered from 6 months, d.c. at 18 months, following serial protocol biopsies and stable LFTs	7/10 (7/7 with non-autoimmune indications) recipients successfully weaned and IS-free for over 6 years	[5,6,125]
Guy’s Hospital, King’s College, UK Safety and efficacy study of Treg therapy in LT patients (ThRIL) (NCT02166177)	Phase I/II	9	Autologous Tregs (TR002). Recipient PBMCs, CD8+-depleted, CD25+-enriched, anti-CD2/CD3/CD28-stimulated cultured with IL-2, SRL. Two dose groups: low, high	TR002 infused as adjunct IS together with ATG, CNI, SRL. Unknown weaning schedule	Completed. No results yet reported	
UCSF, Northwestern, Mayo Clinic, United States Donor alloantigen reactive Tregs for CNI reduction (CTOTC-12) (NCT02474199)	Phase I/II	14	Donor-specific alloantigen reactive Tregs. Publically unknown manufacturing details	Publically unknown IS regimen and weaning procedure	Completed. No results yet reported	
UCSF, United States Donor alloantigen reactive Tregs in LT. (NCT02188719)	Phase I	15	Not publically available. Four-armed study, one control arm, three experimental (of which only one recruited patients)	Cohort 1 (control): ATG+EVR (no Tregs) Cohort 2: cell infusion added (25–60 million cells) Cohorts 3–4: not enrolled. Unknown weaning procedure	Mild AR seen in Cohort 2 only. Enrollment terminated due to several factors: high number of ineligible subjects, slow enrollment, and manufacturing difficulties within the constraints of the funding period	
UCSF, United States Liver transplantation with Tregs (LITTMUS-UCSF) (NCT03654040)	Phase I/II	Target 9	Donor alloantigen-specific Tregs. Recipient leukapheresis to collect PBMC for culture. Manufacturing details not publically available	Single-arm open label study. Tregs given on top of CP (40 mg/kg), CNI to EVR conversion, followed by gradual IS weaning until 52 weeks	Not yet recruiting	
MGH, United States Liver transplantation with Tregs at MGH (LITTMUS-MGH) (NCT03577431)	Phase I/II	Target 9	Single dose of autologous donor alloantigen-reactive Tregs co-stimulatory blockade per protocol (arTreg-CSB)	Single-arm open label study. Tregs given on top of CP (40 mg/kg), CNI to EVR conversion, followed by gradual IS weaning until 52 weeks	Recruiting. No results yet reported	

AR, acute rejection; ATG, antithymocyte globulin; CNI, calcineurin inhibitor; CP, cyclophosphamide; D.C., discontinued; EVR, everolimus; IS, immunosuppression; MMF, mycophenolate mofetil; LFTs, liver function tests; PBMC, peripheral blood mononuclear cells; POD, post-operative day; SRL, rapamycin; LT, liver transplant; SoC, Standard-of-care; Treg, regulatory T-cell; TAC, tacrolimus; DSA, donor specific antibodies; MLR, mixed lymphocyte reaction; TCR, T-cell receptor; DC, dendritic cells; γδ T-cells, gamma delta T-cells, NHP, non-human primate; CKBMT, combined kidney and bone marrow cell transplantation; MC, mixed chimerism; Graft-versus-host disease, GvHD; living donor liver transplantation, LDLT.
